# 12^th ^international conference on human retrovirology: HTLV and related retroviruses

**DOI:** 10.1186/1742-4690-2-61

**Published:** 2005-10-04

**Authors:** Michael D Lairmore, Masahiro Fujii

**Affiliations:** 1 Department of Veterinary Biosciences and Center for Retrovirus Research, The Ohio State University, Columbus, OH 43210 USA; 2 Comprehensive Cancer Center, The Ohio State University, Columbus, OH 43210 USA; 3 Department of Molecular Virology, Immunology, and Medical Genetics, The Ohio State University, Columbus, OH 43210 USA; 4 Division of Virology, Niigata University Graduate School of Medical and Dental Sciences 1-757, Asahimachi-Dori Niigata, Niigata 951-8510 Japan

## Abstract

The 12^th ^International Conference on Human Retrovirology: HTLV and Related Retroviruses, was held at the Half Moon Hotel in Montego Bay, Jamaica, from June 22^nd ^to June 25^th ^2005. The scientific conference, sponsored by the International Retrovirology Association, is held biennially at rotating international venues around the world. The meeting brings together basic scientists, epidemiologists and clinical researchers to discuss findings to prevent HTLV infection or develop new therapies against HTLV-mediated diseases. The Association fosters the education and training of young scientists to bring new approaches to the complex problems of HTLV research, such as translational research to bring findings from the laboratory into clinical trials that benefit HTLV-infected patients. The breadth and quality of research presentations and workshops at the 12^th ^International Conference indicate that these goals are being accomplished. As HTLV research enters its third decade a new generation of scientists face many challenges. However, HTLV scientists and clinicians displayed exciting new approaches and discoveries during plenary talks and poster sessions. The conference encouraged research in HTLV infections and disease, fostered collaborations, and stimulated new partnerships between clinicians and scientists to encourage clinical trials and novel therapeutic interventions.

## Findings

### Jamaica and the International HTLV Conference

The International Retrovirology Association in conjunction with the National Institutes of Health and the University of the West Indies, Jamaica welcomed over 300 scientists from diverse disciplines to the 12^th ^International Conference on Human Retrovirology: HTLV and Related Retroviruses, held at the Half Moon Hotel in Montego Bay, Jamaica, from June 22^nd ^to June 25^th ^2005. The Association was established in May 1994 to promote informal discussions among established human T-lymphotropic viruses (HTLV) scientists to enhance the efforts of scientists and clinicians to form interdisciplinary groups to study HTLV and its related diseases in a cooperative and creative manner. The Association sponsors its biennial general scientific conferences at rotating international venues, generally in areas with endemic HTLV infection. This unique meeting brings together basic scientists, epidemiologists and clinical researchers in a free form exchange of data to discuss approaches to prevent HTLV infection or develop new therapies against HTLV-mediated diseases. The Association has focused its international meeting on HTLV and other related human and nonhuman primate retroviruses, to promote excellent science and to facilitate the communication of scientific results. Equally important, the Association fosters the education and training of young scientists to bring new approaches to the complex problems of HTLV research, such as translational research to bring findings from the laboratory into clinical trials that benefit HTLV-infected patients. Judging from the breadth and quality of research presentations and workshops at the 12^th ^International Conference, these goals are being accomplished.

HTLV research enters its third decade with the challenge to bring a new generation of scientists to the challenges facing the field [1]. Many of the scientists attending the meeting were from developing countries where HTLV is endemic. It was particularly relevant that the conference was held in context to the 25^th ^anniversary of the discovery of the first identified human retrovirus, HTLV-1. Scientists in this field of research have many new discoveries to energize their work, many of which were on display during the meeting as plenary talks or during poster sessions. The conference encouraged research in HTLV infections and disease and fostered collaborations between research groups. Importantly, the conference through its many workshops provided a setting to stimulate new partnerships between clinicians and scientists to encourage the development of clinical trials and novel interventions.

Human T-lymphotropic virus type 1 (HTLV-1) and the closely related HTLV-2 were the first human retroviruses discovered [2]. HTLV-1, is a member of the deltaretrovirus genera, and infects approximately 15 to 20 million people around the world [3]. HTLV-1 infection occurs worldwide, but is particularly endemic in Central Africa, the Caribbean, and South America and southwestern Japan, while HTLV-2 is endemic among Indian tribes of South, Central, and North America. The virus causes adult T cell leukemia/lymphoma (ATLL), an aggressive malignancy of CD4+ T lymphocytes in 1 to 5% of infected individuals that is refractory to most therapies [4]. HTLV-1 is also associated with a progressive neurologic disease termed HTLV-1-associated myelopathy/tropical spastic paraparesis (HAM/TSP) and a variety of chronic inflammatory diseases including infectious dermatitis, uveitis, and arthropathy [5,6]. The following summarizes selected key presentations at the 12^th ^International Conference on Human Retrovirology, but is by no measure a comprehensive overview of all of the exciting findings from the conference and we apologize in advance to those investigators we have not mentioned in an effort to provide a concise summary of the meeting.

## Molecular Epidemiology of HTLV Infections: New Insights

Reports of well characterized cohorts by Dr. Edward Murphy of the University of San Francisco (United States) began the meeting with a clear challenge to carefully examine the relationship between viral loads and disease outcomes. Data from these groups suggest that a "set point" of viral load is determined in each individual studied based on their ability to form an effective early immune response to the infection. Risk factors for the transmission of HTLV-1 from infected mothers to their children based on HLA haplotype was presented by Dr. Robert Biggar and colleagues (National Institutes of Health, United States), who illustrated how immune recognition may determine rates of viral transmission.

A highlight of this session included Dr. Masao Matsuoka's and colleagues' (Kyoto University, Japan) recent findings related to the functional significance of an anti-sense message that codes for the protein HBZ and the role of methylation in the control of HTLV-1 gene expression. His group provided data to indicate that proviral DNA in cell lines derived from ATLL patients was highly methylated correlating with low or absent HTLV-1 gene expression. Unlike the 5' LTR of proviral DNA and the coding region and the 3' LTR was not heavily methylated in these same cells. The expression of HBZ mRNA, but not those encoding other viral genes such as tax, was detected in all the fresh ATL samples, strongly suggesting that HBZ has a significant role in leukemic cell survival in vivo. These findings were further supported subsequently in the meeting by Dr. Eric Wattel (Lyon Rhone, France) who also found correlation between proviral loads in patients with HBZ mRNA expression. In addition, it appears that HBZ expression had a growth stimulating influence on ATL cells. Transfection of HBZ expressing plasmids in an IL-2 dependent human T-cell line augmented the cell line growth and inhibition of HBZ expression by siRNA interference reduced cell growth of ATL-derived cell lines. Finally, transgenic mice containing HBZ gene under the control of a CD4 promoter exhibited an increased number of CD4+ spleen-derived T-cells. Dr. Matsuoka proposed that HBZ and Tax act synergistically to transform CD4+ T-cells eventually leading to the development of ATLL. Since HBZ, but not Tax is expressed in ATL cells in vivo, this unique anti-sense encoded nonstructural protein may represent a promising therapeutic target in ATLL patients. Later in the meeting, the HBZ encoding region was further characterized and mapped to reveal new start sites for the anti-sense transcript by Dr. M. Cavanagh and colleagues (CHUL Research Center, Canada). Collectively, these presentations provide new insights into the complex interaction between HTLV-1 and virus replication and lymphocyte survival.

An exciting summary of recently published findings about novel nucleotide sequences of human retroviruses, HTLV-3 and HTLV-4 were summarized by Dr. William Switzer of the United States Centers for Disease Control and Prevention. These researchers used polymerase chain detection to discover evidence of two related HTLVs (named HTLV-3 and HTLV-4). The first group directed by Switzer investigated 930 central Africans with contact with nonhuman primate blood through hunting and butchering. Serological tests and partial DNA sequencing of blood samples showed that two humans were infected with novel viruses, designated HTLV-3 and HTLV-4. Phylogenetic analysis showed that HTLV-3 is originated from a known monkey virus STLV-3 through cross-species transmission, while a corresponding monkey virus to HTLV-4 has not been reported yet and genetically equidistant from all known HTLVs/STLVs. The second group directed by Dr. Antoine Gessain (Pasteur Institute, France) tested serum specimens or DNA from 240 subjects from Cameroon to provide suggestive evidence that an HTLV-3 related to STLV-3 was present in individuals exposed to nonhuman primate blood or tissues. Importantly, these findings suggest that cross species transmission between nonhuman primates and humans is ongoing where people are exposed to primate blood and tissues [7,8]. A challenge to researchers in the field in the coming years will be to fully characterize these new viruses in terms of complete genomic sequences and to identify replication or pathogenic mechanisms of HTLV-3 and HTLV-4 compared to HTLV-1 and HTLV-2.

## Basic Biology: Novel Mechanisms of Transformation and Disease

The study of HTLV-1 Tax initiated the concept that complex retroviruses regulate their expression by producing transactivating proteins. The mechanisms used by HTLV-1 Tax to alter cell cycle regulation or cell division, as in past meetings, continued to dominate the basic biology sessions of the 2005 meeting. Dr. Jennifer Nyborg (Colorado State University, United States) provided an overview of how the well-studied transactivating protein of HTLV-1, Tax, promotes viral transcription from the chromosomally-integrated HTLV-1 promoter. By using chromatin immunoprecipitation analysis, Dr. Nyborg's group provided data that the promoter activation by Tax1 correlated with the apparent loss of nucleosomes from the promoter and coding region. These results suggest that the Tax functions by causing nucleosome removal from the HTLV-1 promoter. Later in the meeting, by using in vitro transcription using "chromatinized" templates, Dr. Fatah Kashanchi (George Washington University, United States) presented complementary data indicating that Tax converts the randomly-assembled nucleosomes into periodic assembled ones, and this periodic assembly of the nucleosomes correlated with the activation of HTLV-1 promoter by Tax. His group has previously demonstrated that Tax recruits SWI/SNF complex containing BRG1 on the HTLV-1 promoter, and the recruitment is essential for the transcriptional activation of HTLV-1 promoter [9]. Thus, SWI/SNF containing BRG1 may play a role in the reassemble of nucleosomes with the HTLV-1 promoter. Dr. Susan Marriott (Baylor University, United States) provided new data on the effects of HTLV-1 Tax on cell cycle progression and genomic instability. Tax appears to increase genomic instability by altered key checkpoints in the cell cycle. Her work has focused on the G1 and S phases of the cell cycle.

Dr. Kuan-Teh Jeang (National Institutes of Health, United States) presented data that continued this theme of the meeting. His new findings suggest that Tax interacts with the Ran-GTP network to cause abnormal amplification of cellular centrosomes, which may be an initial event of cancer caused by the virus. Dr. Patrick Green (Ohio State University, United States) furthered this subject area by providing new information about the role of the anti-sense encoded protein, PDZ-binding motif of Tax in induction of micronuclei, a hallmark of genomic instability. Dr. Chou-Zen Giam (Uniformed Services University, United States) reported that "unscheduled" activation by the Cdc20-associated anaphase promoting complex by HTLV-1 Tax induces mitotic dysfunction and inactivation of critical regulators of mitosis. This presentations overall solidified the concept that the acquisition of genetic and epigenetic changes in Tax-expressing T cells favors the selection of cells with mutated proviral DNA in a manner to favor eventual outgrowth of the transformed cell in a way that avoids immune system recognition. Collectively, the precise role of Tax in cellular transformation was a major theme of the basic science sections of the meeting.

Dr. Warner Greene (Gladstone Institute, United States) provided a stimulating plenary talk about the role of APOBEC 3G (A3G), a cellular deoxycytidine deaminase with broad anti-retroviral activity. Dr. Greene summarized what is currently understood about A3G, which is inactive as a high-molecular-weight ribonucleoprotein complex in activated CD4 T-cells, but is active in low-molecular-weight complexes in resting CD4 T-cells. Resting CD4+ T-cells are typically resistance for HIV-1 infection, but the resistance was greatly relieved by A3G-specific siRNAs that block A3G function. Surprisingly, sequence analysis showed rare dG-dA hypermutations, a signature of A3G-mediated inhibition, in infected CD4 T-cells. Thus, A3G may have another mechanism to inhibit HIV infection. Dr. Greene suggested that deaminase activity may have a broader role and perhaps may influence HTLV-1's ability to infected CD4+ T-cells, a preferred natural target of HTLV-1 infection in vivo. In this regard, Dr. David Derse and colleagues (National Cancer Institute, United States) at the meeting reported that exogenous overexpression of APOBEC 3B (A3G) also inhibited HTLV-1 infectivity. A3G was incorporated into HTLV-1 virions, but the amount was much less than that of HIV-1 stains that lack the ability to inactive the cellular co-factor (HIV Vif defective). Interestingly, HTLV-1 does not appear to have *vif*-like gene in its genome, but data presented from Dr. Derse suggested that the HTLV-1 structural gene, *gag *contained sequences that act to inhibit A3G incorporation into HTLV-1 virions. Thus, HTLV-1 may overcome A3G anti-retroviral activity through *gag*-mediated exclusion of A3G. Consistently with these results are findings that indicate that there are rare dG-dA hypermutations among HTLV-1 isolates.

## Clinical and Translational Research: New Approaches in Therapy

The meeting brought together a variety of clinicians seeking better treatments against ATLL and HAM/TSP. Dr. Thomas Waldmann (National Institutes of Health, United States), a pioneer in the field of therapeutic approaches against ATLL, provided a summary of the role of IL-15 in T-cell signaling and his groups recent findings involving Hu-Mik-Beta-1 (HM-beta) to block β-chain signaling following IL-15 receptor engagement. This approach may provide exciting new avenues to inhibit lymphocyte-mediated disorders such as HAM/TSP. Clinical trials using the potent immunosuppresive agent Campath-1H (anti-CD52) was presented by Dr. J.C. Morris (National Institutes of Health, United States). HTLV-1 Leukemic patients appeared to respond well to Campath-1H, despite its adverse side effects. A key factor in non-responsive ATL lymphoma patients appears to be the difficulties with drug penetration of solid tissues.

HTLV-1 Tax has been shown to interact directly with different members of the NF-κB family [10]. Interference with post-translational modifications of Tax including ubiquitylation and sumoylation by proteasome inhibition was presented by Dr. Ali Bazarbachi and colleagues (American University, Beirut, Lebanon) as effective ways to block NF-κB signaling, a key pathway of Tax-mediated cell transformation. Dr. Lee Ratner (Washington University, United States) presented new data regarding his novel transgenic mouse model. His new work indicates from this model indicates that both the canonical and non-canonical pathways of NF-κB activation are involved in resistance to apoptotic stimuli in Tax transgenic mouse derived cell lines.

Novel treatments continue to be a focus of researchers in the HTLV and related retrovirus field. Dr. Luc Willems and colleagues (Faculte Universitaire des Sciences Agronomiques, Belgium) using their established bovine leukemia virus infection of sheep model, revealed new approaches to treatment of leukemia using the drug, valproate. This treatment was shown to be effective in decreasing lymphocyte numbers and tumor regression in this model system.

## Educational and Technical Workshops: Bringing Scientists together to Address Complex Problems

A particularly interactive portion of the meeting were the educational and technical workshops held offsite at the Barnett Estates, a serene setting that promoted scientific exchange. Morning workshops discussed the epidemiology, animal models, immunology, and basic virology of HTLV and related viruses. These group sessions were spirited and were lead by scientist who provided an excellent overview of their fields before leading the discussion of selected topics of interest to the group. These sessions allowed a more informal exchange of ideas and unpublished data, a goal of any scientific meeting. New virus strains including HTLV-3 and HTLV-4 characterized by molecular signatures were the subject of many conversations. Current strengths of weakness or gaps in the literature dominated many sessions as participants provided their interpretation of recent published or new data at the early plenary sessions. These workshops were extended in the afternoon to include important and clinically focused sessions based on HTLV disease associations. Thus, workshops devoted to adult T-cell leukemia/lymphoma, infectious dermatitis, and HTLV-1 neurologic disease allowed scientist to debate current approaches and how clinical trials could be evaluated and improved. Overall, these workshops provided a dynamic interaction among scientists and clinicians, which were apparent when patient case studies were included to focus the discussions.

## Viral Pathogenesis: Interplay between HTLV's and Lymphocytes

Dr. Masahiro Fujii and colleagues (Niigata University, Niigata, Japan) reported data using co-transfection assays of Tax expression plasmids with luciferase reporters to test the functional differences between HTLV-1 and HTLV-2 Tax [11]. This group's results suggest that IL-2 autocrine secretion establishes benign life-long HTLV-2 infection and the distinct cytokine production regulated by the nuclear factor of activated T-cells (NFAT) is a key factor for the distinct differences in disease association between these two related viruses. Further insights into the pathogenesis of HTLV-1 infection was provided by Dr. Brian Wigdahl (Drexel University, United States) who provided provocative data indicating that Tax is not only secreted from infected cells, but can differentially modulate the function of dendritic cells and astrocytes. This work may provide a unique mechanism of neurologic damage by HTLV-1. Dr. Renaud Mahieux and colleagues (Institut Pasteur, France) continued to implicate unique molecular features that differentiate HTLV-1 Tax (Tax-1) from HTLV-2 Tax (Tax-2). Tax-2 appears to have a distinct cytoplasmic distribution compared to Tax-1. Futures studies to define the role of particular motifs that may explain the pathogenic differences between HTLV-1 and HTLV-2 will be directed at these important regulatory proteins.

Dr Claudine Pique and colleagues (Saint Louis Hospital, Paris, France) presented new findings that neuropilin 1 (NP-1), a receptor for Sematophorin 3a and vascular endothelial growth factor (VEGF), acts as a co-factor for HTLV-1 entry. NP-1 directly interacted with HTLV-1 envelope protein. There appears to be two separable domains in the HTLV-1 envelope that are required for the virus entry. While one domain is a binding surface for GLUT-1, a recently identified HTLV-1 receptor, the other was that for NP-1. NP-1, GLUT-1 and the envelope proteins appear to form a ternary complex on the cell surface. These results argued that HTLV-1 has two cellular receptors for the viral entry into host cells similar to HIV-1.

Dr. Steven Jacobson (National Institutes of Health, United States) presented new information about the intriguing findings related to regulatory T-cells (CD4+, CD25+, Foxp3+) in the immunopathogenesis of HTLV-1-associated neurologic disease. Interestingly, his research group found that Tax specifically inhibits *foxp3 *expression, which would be predicted to suppress the function of these important regulatory T-cells, perhaps contributing to lymphocyte-driven diseases. Dr. Charles Bangham (University of London, United Kingdom) and his research group continue to lead the field of cellular immunity against HTLV-1 infection. His presentation provided novel insights into the efficiency of cytotoxic T-cell killing as a key determinant of patient viral load outcome and data describing unique lymphocyte labeling techniques (6,6-D(2)-glucose) to monitor the kinetics of lymphocyte proliferation and death in HTLV-1-infected subjects.

The role of nonstructural proteins including proteins included in pX ORFs 1 and 2 were featured by a number of investigators. Dr. Franchini's research group (National Cancer Institute, United States) presented work indicating that p12^I ^is recruited to the immunological synapse and inhibits signaling from the T-cell receptor. Dr. Vincenzo Ciminale (University of Padova, Padova, Italy) provided an update of his research of a novel mitochondrial localizing protein p13^II ^in modifying lymphocyte survival and apoptotic cell death. Dr. Michael Lairmore and his colleagues (Ohio State University, United States) provided new information on the role of p30^II ^in modifying G2 exit of the cell cycle furthering implicating this protein in lymphocyte survival.

## Conclusions and Perspective

The meeting concluded with poignant remarks by one of the pioneering epidemiologist in the field of HTLV research, Dr. Nancy Mueller (Harvard, United States). She provided appropriate context to past studies of cohorts of HTLV-1 infected subjects, disease associations, and the useful applications of carefully controlled population-based studies. As the meeting came to a close, it was clear that HTLV and related retrovirus researchers faces many challenges, which confound and frustrate scientists in the HTLV field. However, the 12^th ^International Conference on Human Retrovirology illustrated the dedication of the many scientists, clinicians, and patient advocates to address these challenges with a new determination and energy, as they all reluctantly left the gracious atmosphere of Jamaica to travel back to their homes, laboratories and offices throughout the world.

## List of Abbreviations

HTLV, human T-lymphotropic viruses

HTLV-1, human T-lymphotropic virus type 1

HTLV-2, human T-lymphotropic virus type 2

HAM/TSP, HTLV-1-associated myelopathy/tropical spastic paraparesis

ATLL, adult T-cell lymphoma/leukemia

## Competing interests

The authors have no competing financial or other interests involved in the data, methods, or writing of this manuscript.

## Authors' contributions

Each author (MF and ML) have each met the definition of author as outlined by the Retrovirology journal. Each has made substantive intellectual contributions to the commentary. Each author has given final approval of the version to be published. Each author has participated sufficiently in the work to take public responsibility for appropriate portions of the content.
